# National Trends in Healthcare-Associated Infections and Evaluation of Infection Prevention and Control Policies in Uzbekistan (2015–2024)

**DOI:** 10.3390/healthcare14193161

**Published:** 2026-09-24

**Authors:** Nilufar T. Khamzaeva, Bakhodir B. Rakhimov, Ibrohim K. Mamatkulov, Farangiz O. Abdullaeva, Muattarkhon F. Abdukakharova, Malika O. Kurbaniyazova

**Affiliations:** 1 Department of Epidemiology, Tashkent State Medical University, Tashkent 100109, Uzbekistan; m.abdukaxarova@tashmeduni.uz (M.F.A.); m.kurbaniyazova@tashmeduni.uz (M.O.K.); 2 Department of Environmental Hygiene, Tashkent State Medical University, Tashkent 100109, Uzbekistan; b.b.raximov@tashmeduni.uz; 3 L.M. Isaev Research Institute of Microbiology, Virology, Infectious and Parasitic Diseases, Samarkand State Medical University, Samarkand 100400, Uzbekistan; bibinor@list.ru; 4 English Language Department, Kimyo International University in Tashkent, Tashkent 100121, Uzbekistan; f.abdullaeva@kiut.uz

**Keywords:** cross infection, infection control, Uzbekistan, population surveillance, health policy, drug resistance, microbial

## Abstract

**Highlights:**

**What are the main findings?**

- Reported national HAI incidence fell 45.3% between 2015 and 2024, from 3.95 to 2.16 per 100,000 population (Mann–Kendall *p* = 0.007); surgical site infections were the most frequently reported HAI type (44.1%).
- Burden was uneven across regions, and ranking by incidence, rather than case count, reordered the national picture.

**What are the implications of the main findings?**

- Case-finding is passive and paper-based, so the decline cannot be separated from a possible fall in detection.
- Eight corrective actions are proposed, each tied to a WHO Core Component.

**Abstract:**

**Background/Objectives:** Healthcare-associated infections (HAIs) disproportionately burden low- and middle-income countries, but no such description has been published for Uzbekistan. We describe national trends in reported HAIs over 2015–2024 with their regional and clinical distribution and review the country’s infection prevention and control (IPC) regulations against the eight World Health Organization (WHO) Core Components. **Methods:** We analysed the complete national series of reported HAI cases for 2015–2024 (ten annual counts; 817 cases in 2024, disaggregated by region and HAI type), with official population denominators. Trend was assessed by the Mann–Kendall test, log-linear average annual percentage change (AAPC) and the Theil–Sen slope estimator, with 2020 treated separately as pandemic-affected. The five binding national IPC instruments in force were reviewed against each Core Component using five pre-specified criteria. **Results:** Reported incidence fell from 3.95 to 2.16 per 100,000 population (45.3%; Mann–Kendall *p* = 0.007; Theil–Sen slope −0.186 per 100,000 per year, 95% confidence interval (CI) −0.293 to −0.110). The fall in 2020 was isolated (1.00), with an underlying decline of about 6% per year otherwise (AAPC −6.36%, 95% CI −8.34 to −4.33). Tashkent city accounted for 31.7% of cases and had the highest incidence (8.19 per 100,000) and bed density; Jizzakh (3.55) and Navoi (3.44) had elevated incidence at close to average bed density. Surgical site infections were the most frequently reported HAI type (44.1%). Of the eight Core Components, one met all five criteria, six met some and one none; verification was the most frequent shortfall, absent for seven of eight. **Conclusions:** This is the first decade-long description of reported HAIs in Uzbekistan. Incidence declined substantially, but passive case-finding and the 2020 disruption mean the data cannot separate a fall in occurrence from a fall in detection. Standardised case definitions and facility-level denominators are the first priority among the eight corrective actions proposed.

## 1. Introduction

Healthcare-associated infections (HAIs) are infections acquired during healthcare that were not present or incubating at admission [1]. The World Health Organization (WHO) estimates that about 7% of patients in high-income countries and up to 10% of patients in developing countries acquire at least one HAI during hospitalisation. The burden in low- and middle-income countries (LMICs) is several times higher, and surgical site infections (SSIs) are typically the most prevalent type [2,3,4]. HAIs prolong hospital stay by 7–15 days, increase treatment costs two- to three-fold and raise mortality, exceeding 50% in intensive care [2,3]. Gram-negative organisms account for an estimated 32% of HAI aetiology worldwide and *Staphylococcus aureus* for 15–25%, with resistant *Enterobacterales* being of particular concern given limited treatment options [5,6].

HAIs and antimicrobial resistance (AMR) are interlinked. The WHO 2024 Global Report on Infection Prevention and Control attributes an estimated 75% of the global AMR burden to HAIs, and projects that better infection prevention and control (IPC) in LMIC settings could avert at least 337,000 AMR-associated deaths each year [7,8]. Strengthening national HAI surveillance is therefore a foundational AMR-containment strategy.

Empirical HAI data from Central Asia remain sparse. A Kazakhstani intensive care study (Astana, 2014–2015) found Gram-negative pathogens predominant and no functioning regional surveillance system [9]. A 2022 pilot point-prevalence survey in four Kazakhstani tertiary hospitals, using European Centre for Disease Prevention and Control (ECDC) methodology, found an active HAI in 3.8% of patients [10]. A nationwide 2023 survey of 26 hospitals and 8076 patients found an active HAI in 2.4% of patients [11]. For Uzbekistan, we identified neither a published description of HAI epidemiology nor any assessment of the regulatory framework against the WHO *Guidelines on Core Components of Infection Prevention and Control Programmes* (2016) [12].

Several facets of IPC provision are lacking in Uzbekistan. No nationwide electronic or real-time HAI surveillance system has been described, and standardised case definitions of the kind used internationally are not in national use. Nor has it been documented how far the national digital health platform supports HAI case detection. We therefore describe trends in reported HAIs over 2015–2024, with their regional and clinical distribution, and review the IPC regulations against the WHO Core Components to place those findings in policy context.

## 2. Materials and Methods

**Data sources.** We analysed the complete national series of officially reported HAI cases for Uzbekistan, 2015–2024, obtained from the State Sanitary-Epidemiological Surveillance Centres [13]. The series comprises ten annual aggregate counts; the 2024 total (817 cases) is additionally disaggregated by region and HAI type. No sampling was performed. Cases are ascertained through passive, notification-based surveillance: ward staff submit a standard emergency notification form (F.085/U) for each suspected HAI, which is subsequently coded to the International Classification of Diseases, 10th revision (ICD-10) by the Surveillance Centres. We are not aware of a routine independent validation step applied to these notifications and treat this as a data-quality limitation. Official annual population estimates from the National Statistics Committee [14] served as denominators, and regional healthcare infrastructure indicators were obtained from the Statistics Agency under the President of the Republic of Uzbekistan [15].

**Statistical analysis.** Incidence rates were calculated per 100,000 population using official annual estimates; a population-based denominator was used because cases are notified at population level and no admissions or patient-day denominator exists nationally. Regional and HAI-type distributions for 2024 are expressed as counts and proportions of the national total, with region-specific incidence calculated from region-level counts and denominators [13,14]. Monotonic trend was assessed with the Mann–Kendall test. AAPC was derived from the slope (*β*) of a log-linear regression of the annual rate on calendar year, as (e*^β^* − 1) × 100, with the 95% confidence interval (CI) taken from the standard error of the slope. Because 2020 was judged in advance to be pandemic-affected, AAPC was estimated both with and without that year. Trend magnitude was also quantified with the Theil–Sen slope estimator (sens.slope, R package trend). Segmented and Poisson models were not fitted, given only ten annual points. Analyses were conducted using R 4.3.3 (R Foundation for Statistical Computing, Vienna, Austria) and cross-validated against an independent Python 3.14.3 (Python Software Foundation, Wilmington, DE, USA) and SciPy 1.18.1 implementation; *p* < 0.05 (two-sided) was considered significant.

**Review of national IPC regulations.** HAI prevention in Uzbekistan is governed by five legally binding national instruments, all of which were in force on 15 June 2026, and all of which were reviewed: SanPiN No. 0342-17 (sanitary-hygienic, preventive and anti-epidemic requirements for healthcare facilities) [16]; SanPiN No. 0317-15 (medical waste) [17]; Ministry of Health Order No. 92 of 2019 (Infection Control Committee and staff training) [18]; SanQvaN No. 0020-22 (facility design and room air cleanliness) [19]; and Methodical Guideline No. 06-11 (IPC monitoring and audit) [20]. Instruments were identified through the national legal information portal (lex.uz) and official ministry channels; non-binding guidance and instruments addressing HAIs only incidentally were excluded. For each of the eight WHO Core Components [12], every corresponding provision was evaluated against five pre-specified criteria: corresponding scope, mandatory status, quantified specification, universal facility coverage and the existence of a verification mechanism. These map onto three categories of alignment: full, partial, or weak. Two authors (N.T.K., B.B.R.) applied the criteria independently and blinded, agreeing on six of eight Core Components (Cohen’s *κ* = 0.53), then reviewed all eight jointly against the recorded clauses. The classifications describe what the regulations require, not what facilities do; the criteria, decision rule and clause-level record are given in Supplementary Tables S3 and S4.

**Literature search and ethics.** We searched PubMed and Google Scholar (last searched 15 June 2026; no start-date restriction; English and Russian records) combining “healthcare-associated infection”, “nosocomial infection”, “Uzbekistan”, “surveillance”, “infection prevention and control”, “WHO Core Components” and “Infection Prevention and Control Assessment Framework (IPCAF)”. This study used aggregated, de-identified surveillance statistics and public regulatory documents; no individual patient data were involved.

## 3. Results

### 3.1. National Trends, 2015–2024

Between 2015 and 2024, the reported national HAI incidence rate declined from 3.95 to 2.16 per 100,000 population, a 45.3% reduction (Table 1) [13]. A pronounced decline occurred in 2020 (1.00 per 100,000), plausibly reflecting pandemic-related disruption of admissions and surveillance capacity, although no facility-level admissions or notification records for 2020 were available to test this. Between 2021 and 2024, the rate stabilised between 2.1 and 2.5 per 100,000.

The Mann–Kendall test indicated a significant decreasing trend across the full series (*S* = −31, *Z* = −2.68, *p* = 0.007) and, more consistently, excluding 2020 (*S* = −30, *Z* = −3.02, *p* = 0.002). The Theil–Sen slope was −0.186 per 100,000 per year for the full series (95% CI −0.293 to −0.110) and −0.186 (95% CI −0.250 to −0.134), excluding 2020. Log-linear AAPC was −6.94%/year for the full series (95% CI −14.88 to 1.75; *R*^2^ = 0.30; *p* = 0.10), the wide interval reflecting the 2020 outlier. Excluding 2020, AAPC was −6.36%/year (95% CI −8.34 to −4.33; *R*^2^ = 0.88; *p* < 0.001). All three estimators agree on an underlying decline of approximately 6–7% per year, on which 2020 was superimposed (Figure 1).

### 3.2. Regional Distribution, 2024

The regional distribution in 2024 was markedly heterogeneous (Table 2, Figure 2). Tashkent city accounted for the largest proportion of reported cases (259; 31.7%), followed by Samarkand (9.1%) and Andijan (8.0%); the lowest proportions were in Bukhara and Namangan.

Because proportions of the national total are inflated by regional population size independently of true risk, we also calculated region-specific incidence (Table 2). Ranking by incidence confirms Tashkent city as the highest-burden region but reorders the rest. Jizzakh (3.55) and Navoi (3.44) rank second and third despite ranking fifth and ninth by case proportion, while Samarkand and Andijan fall to ninth and seventh. Tashkent city’s bed density (81.9 per 10,000) exceeds every other region by 55–164% (range 31.0–52.9; national average 47.4), consistent with its tertiary-care facilities. Jizzakh and Navoi are close to average (44.0 and 41.8), so their elevated incidence is unlikely to reflect healthcare capacity and warrants local investigation.

### 3.3. Classification of HAIs, 2024

Surgical site infections were the most frequently reported HAI type in 2024 (44.1%; Table 3), followed by puerperal (16.5%) and neonatal (15.5%) purulent-septic conditions (Figure 3). The registry (Form F.085/U) does not disaggregate SSIs by specialty or procedure, so the operations contributing most cannot be identified; internationally, abdominal, obstetric and orthopaedic procedures are the largest contributors in LMIC settings [3,4]. Over 2015–2024, the rate of acute upper respiratory tract infections fell 6.8-fold and that of healthcare-associated hepatitis B 5.7-fold [13], in contrast to the stable proportion of surgical site infections.

### 3.4. Alignment of National IPC Regulations with the WHO Core Components

Applying the five criteria produced the classifications in Table 4; the clause-level record supporting every cell is given in Supplementary Table S4.

Two patterns emerge. Only CC8 met all five criteria; six Core Components met some but not all, and CC7 met none. The shortfall is also uneven across criteria: verification was absent for seven of eight Core Components, corresponding scope for three, universality for two and quantification for two. The characteristic weakness is therefore not the absence of rules but the absence of any mechanism to confirm that mandated rules are performed.

## 4. Discussion

Reported HAI incidence declined over 2015–2024 alongside a regulatory framework that is broad in coverage but rarely verifiable. We compare the IPC components that are lacking with two comparators: Kazakhstan, the closest Central Asian setting with recent ECDC-methodology survey data, and Romania, whose 20-year legislative analysis documents a similar gap between regulatory design and implementation.

### 4.1. Interpreting the Incidence Trend

The sharp 2020 decline is not read as genuine improvement; pandemic-related disruption to admissions and reporting is more plausible, though we could not test it. The steadier decline of approximately 6–7% per year outside 2020 is present both before and after that year and cannot be explained by pandemic disruption. Two explanations fit, and our aggregate data cannot separate them. The strengthening of regulatory and training infrastructure across the decade [18] may have improved practice, consistent with the declines in acute respiratory infections and hepatitis B. Alternatively, passive notification may have gradually lost case ascertainment, so part of the decline may reflect falling detection.

The reported incidence is orders of magnitude below the HAI prevalence figures usually cited internationally (commonly 7–10% of hospitalised patients), but this gap is expected. The Uzbek figures are population-based incidence from passive notification, whereas internationally cited percentages are point-prevalence or admission-based estimates from active, criteria-based case-finding. The low absolute incidence reflects the sensitivity of the surveillance architecture at least as much as the occurrence of disease.

### 4.2. Surgical Site Infections and Multimodal Strategies

The predominance of SSIs (44.1%) is consistent with international LMIC experience [3,4] and connects directly to the CC5 finding. WHO guidance treats hand hygiene, disinfection, sterilisation and isolation as one coordinated strategy [12,21], whereas the Uzbek texts regulate them separately. A 66-country cohort reported a 30-day SSI incidence of 12.3%, rising to 23.2% in low-human-development-index settings [22,23], and a multimodal bundle across five African hospitals reduced incidence from 8.0% to 3.8% [24]. Kazakhstani surveys found a similar proportion (42.9% of ward HAIs) [10,11], supporting a genuine regional priority rather than a surveillance artefact.

### 4.3. Training, Staffing, Surveillance and Audit

Order No. 92 specifies a mandatory 36 h curriculum [18] but no competency assessment, so translation into practice cannot be confirmed. The same pattern is reported elsewhere, where formally present IPC provisions lacked the specificity needed to change behaviour [25,26]. A dedicated epidemiologist is mandatory only above 200 beds, and nurse staffing, workload and bed-occupancy standards are absent. These are outcome-relevant: low nurse-to-patient ratios were independently associated with higher HAI and carbapenem-resistance rates [27], and IPC staff in LMICs report workload pressure and limited dedicated time [28].

Case ascertainment relies on paper-based notification and ICD-10 logbooks, without standardised case definitions or a real-time electronic module. Infection-control committees, though universally mandated and meeting quarterly, operate without indicator-based monitoring or structured feedback [18,20], the same pattern being identified in a Kazakhstani intensive care study [9]. Order No. 270 (2025) digitised many facility records into the national digital health information system (DMED) platform, but it includes no dedicated module for HAI case-finding [29]. This gap is shared by most LMICs, only 16% of which had national or sub-national HAI surveillance as of 2010 [30,31]. A multicentre Indian initiative adapted National Healthcare Safety Network (NHSN)/ECDC case definitions to locally available laboratory data rather than awaiting a bespoke national standard [30], with syndromic case-finding as a lower-cost interim option [32]. Supplementary Tables S1 and S2 situate the Uzbek model internationally. The Romanian analysis cautions against treating regulatory alignment as an endpoint, since formal alignment with EU standards did not resolve workforce and reporting-culture barriers [33]. Passive surveillance also limits detection of resistant-organism clusters, as well as of cases, compounding the linked AMR burden [34].

The single full classification is instructive: the waste, air cleanliness and sterilisation-zoning requirements are quantified and, uniquely, paired with a verification mechanism [20]. A system able to draft and verify rules at this level of detail is not short of regulatory capacity; the gap lies in extending that approach to the personnel and electronic infrastructure on which surveillance depends.

### 4.4. Policy Implications

Table 5 sets out the corrective action corresponding to each identified deficiency.

### 4.5. Limitations

Our analysis relies on officially reported cases, and given well-documented under-ascertainment internationally [3,35,36], the true burden is likely higher; no instrument mandates active in-hospital case-finding, and we cannot confirm that notification practices remained consistent across 2015–2024. The population-based denominator is not numerically comparable with the facility-level denominators used internationally; with ten annual points, we could not fit segmented or Poisson models; and regional and HAI-type data were available for 2024 only. The regulatory review assesses what the regulations require rather than what facilities do and uses a study-specific set of criteria rather than IPCAF [37]. It rests on eight units, so the reported *κ* describes the initial independent round rather than the precision of the final classifications. Independent external re-coding and a national IPCAF application are the appropriate next steps [38,39].

## 5. Conclusions

This study provides the first decade-long description of HAI incidence in Uzbekistan, alongside the first review of its IPC regulations against the WHO Core Components. Reported HAI incidence fell by 45.3% between 2015 and 2024. The 2020 disruption and the limits of passive notification mean that these data cannot distinguish a reduction in occurrence from a reduction in detection, and the low absolute rates are not evidence of a low true burden. Burden was uneven across regions in a way only partly explained by healthcare capacity, and surgical site infections predominated throughout. The national framework proved comprehensive in coverage but rarely verifiable, and we propose eight corrective actions, of which standardised case definitions and facility-level denominators are the immediate priority.

## Figures and Tables

**Figure 1:**
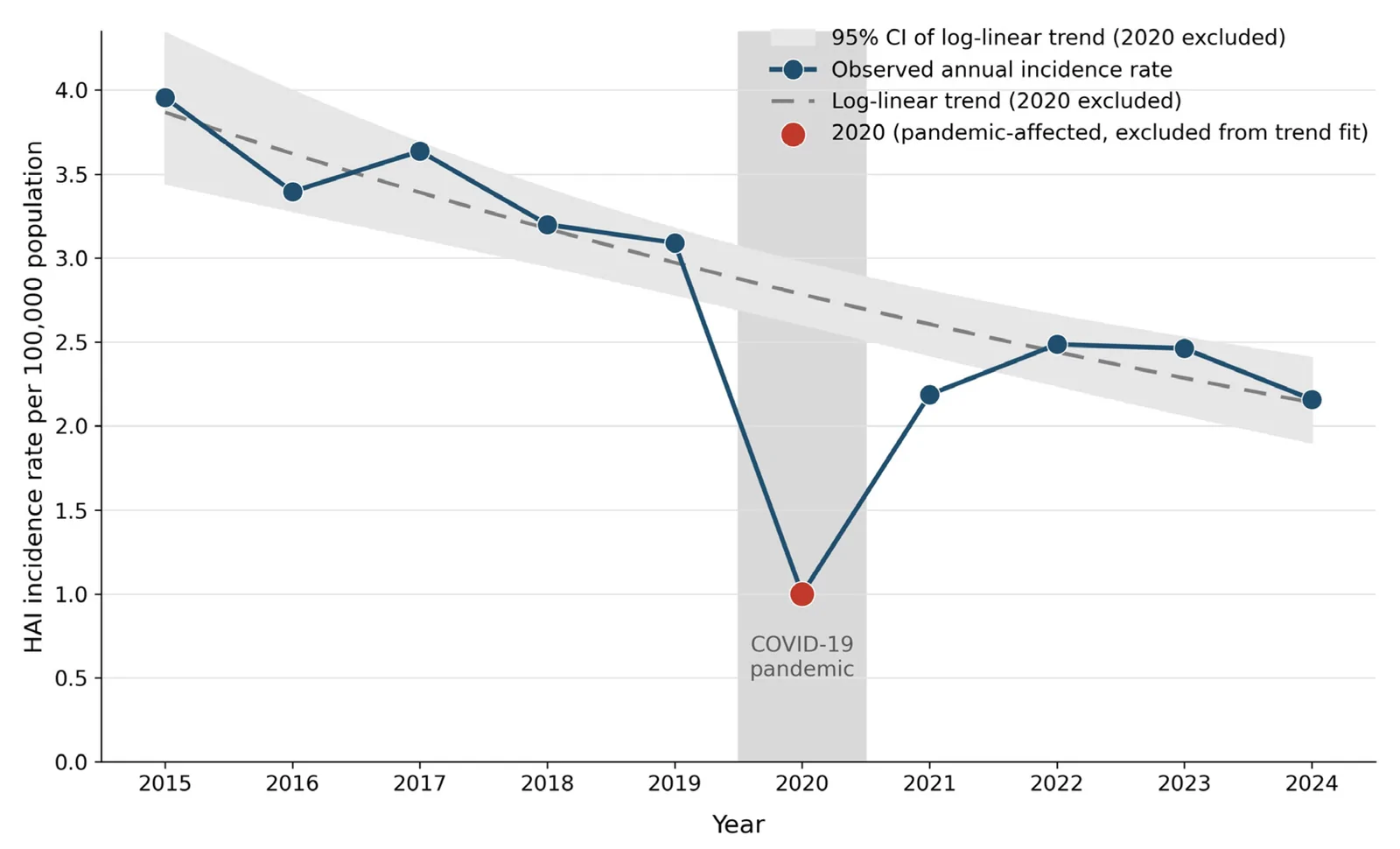
Reported national HAI incidence rate, Uzbekistan, 2015–2024 (per 100,000 population). The solid line and circles show the observed annual incidence rate. The 2020 point (highlighted) was excluded from the trend fit owing to pandemic-related disruption. The dashed line shows the log-linear trend, excluding 2020 (AAPC = −6.36%/year; 95% CI −8.34 to −4.33; R^2^ = 0.88; *p* < 0.001); the shaded band shows the 95% confidence band.

**Figure 2:**
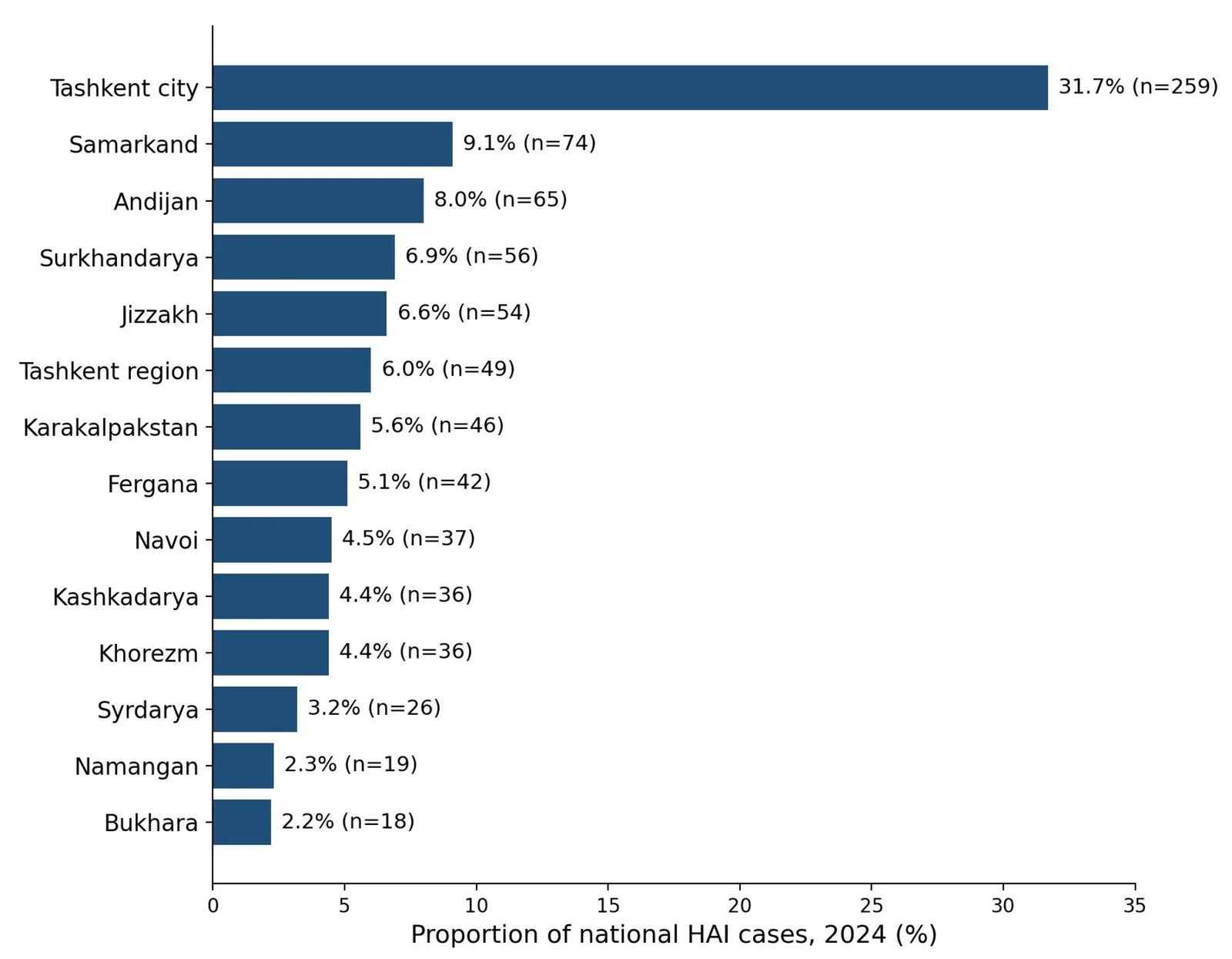
Regional distribution of reported HAI cases, Uzbekistan, 2024. Bars show each region’s proportion of the national total (%) with case counts (n) labelled, ranked from highest to lowest.

**Figure 3:**
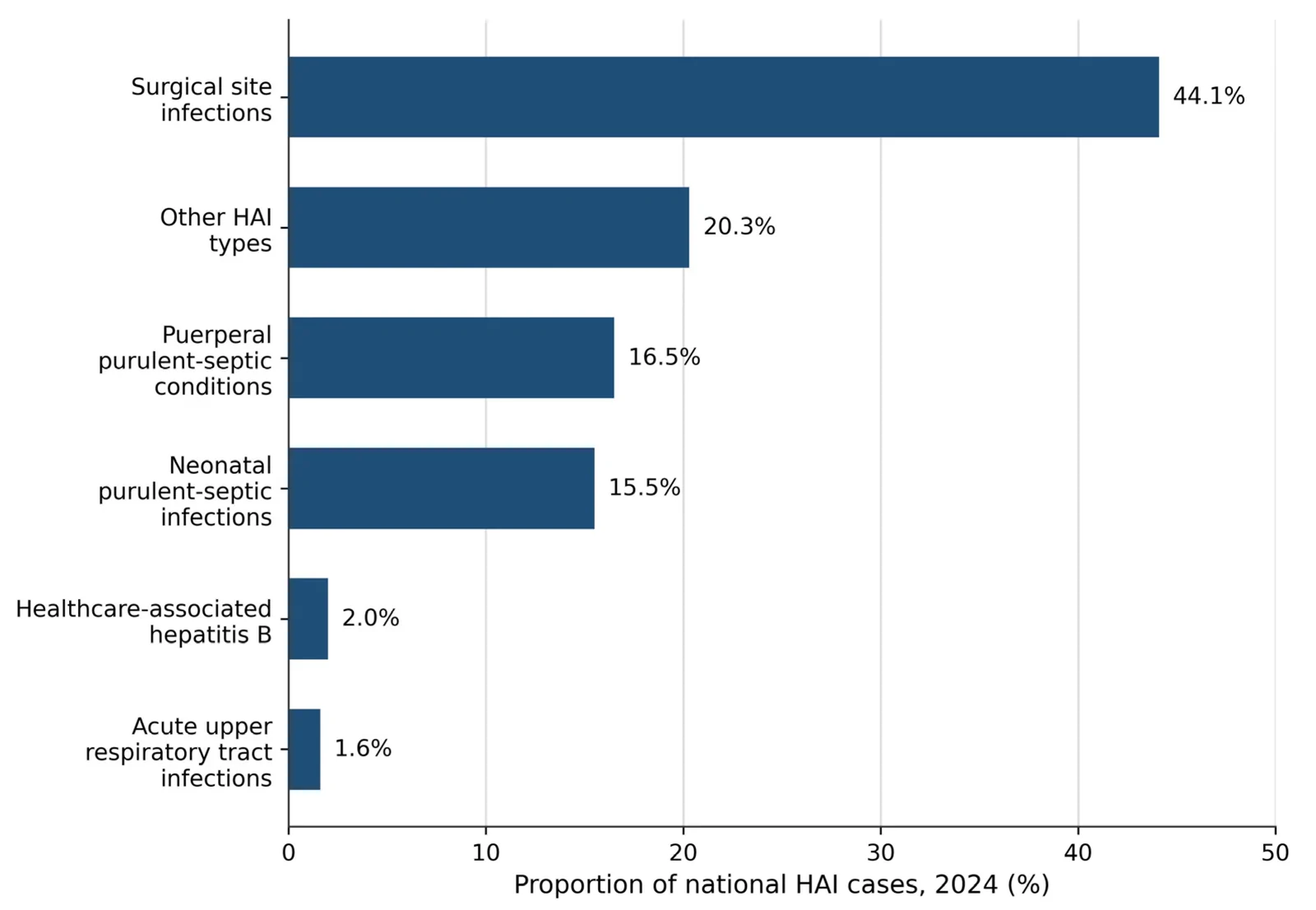
Classification of reported HAI cases, Uzbekistan, 2024 (proportion of the national total, %). “Other HAI types” (20.3%) is an aggregate of the remaining, individually less frequent HAI types not separately itemised in the source data.

**Table 1:** Reported national healthcare-associated infection (HAI) incidence rate in Uzbekistan, 2015–2024 (per 100,000 population) [13].

Year	Reported HAI Incidence Rate per 100,000 Population
2015	3.95
2016	3.40
2017	3.64
2018	3.20
2019	3.09
2020	1.00
2021	2.19
2022	2.49
2023	2.46
2024	2.16

**Table 2:** Regional distribution, population-standardised incidence and hospital-bed density of reported HAI cases, 2024.

Region	Cases, n	Proportion, %	Incidence, per 100,000 Pop.	Hospital Beds, per 10,000 Pop. [15]
Tashkent city	259	31.7	8.19	81.9
Samarkand region	74	9.1	1.71	39.5
Andijan region	65	8.0	1.86	45.2
Surkhandarya region	56	6.9	1.90	33.6
Jizzakh region	54	6.6	3.55	44.0
Tashkent region	49	6.0	1.49	52.9
Republic of Karakalpakstan	46	5.6	2.35	41.9
Fergana region	42	5.1	1.01	41.8
Navoi region	37	4.5	3.44	41.8
Khorezm region	36	4.4	1.77	39.3
Kashkadarya region	36	4.4	0.97	31.0
Syrdarya region	26	3.2	2.89	52.2
Namangan region	19	2.3	0.62	51.2
Bukhara region	18	2.2	0.87	44.9
**Total**	**817**	**100.0**	see Table 1	**47.4**

**Table 3:** HAI type distribution, 2024.

HAI Type	n	%
Surgical site infections	360	44.1
Puerperal purulent-septic conditions	135	16.5
Neonatal purulent-septic infections	127	15.5
Healthcare-associated hepatitis B	16	2.0
Acute upper respiratory tract infections	13	1.6
Other HAI types	166	20.3
**Total**	**817**	**100.0**

**Table 4:** Alignment of the Uzbek IPC regulatory framework with the WHO Core Components. Criteria: S = corresponding scope; M = mandatory; Q = quantified; U = universal facility coverage; V = verification mechanism. ✓ = met; ✗ = not met.

CC	WHO Core Component	Key National Provision	S	M	Q	U	V	Alignment
CC1	IPC programme	Committee in every facility; epidemiologist only above 200 beds [18]	✓	✓	✓	✗	✗	Partial
CC2	IPC guidelines	National guideline in force; no revision or audit cycle [16]	✓	✓	✓	✓	✗	Partial
CC3	Education and training	36 h, 12-topic curriculum; no competency assessment [18]	✓	✓	✓	✓	✗	Partial
CC4	HAI surveillance	Paper notification with ICD-10 coding; no case definitions [16]	✗	✓	✗	✓	✗	Partial
CC5	Multimodal strategies	Hygiene, disinfection, sterilisation, isolation regulated separately [16]	✗	✓	✓	✓	✗	Partial
CC6	Monitoring, audit, feedback	Committee meets at least quarterly; bacteriological monitoring [18,20]	✓	✓	✓	✓	✗	Partial
CC7	Workload, staffing, bed occupancy	None; the 200-bed threshold is counted under CC1	✗	✗	✗	✗	✗	Weak
CC8	Built environment and equipment	Waste classes, air cleanliness classes, zoning, with monitoring [17,19,20]	✓	✓	✓	✓	✓	Full

**Table 5:** Identified deficiencies by Core Component and corresponding corrective actions.

CC	Criterion Not Met	Specific Deficiency	Recommended Action	Priority
CC4	Q	No facility-level denominators	Mandate collection of admissions, patient-days, device-days, procedures, deliveries, and neonatal admissions	1
CC4	S	Passive notification only	Pilot sentinel active surveillance in tertiary hospitals for SSI, CAUTI, CLABSI, VAP, and neonatal sepsis using CDC/NHSN- or ECDC-aligned case definitions	2
CC4	V	No electronic HAI module in DMED	Add an HAI module flagging probable cases from microbiology, antibiotic prescriptions, fever records, re-operation, prolonged stay and readmission	3
CC1, CC7	U (CC1); S (CC7)	Epidemiologist only above 200 beds; no staffing standard	Extend mandatory IPC staffing below 200 beds via regional shared-epidemiologist teams; introduce nurse staffing and bed-occupancy standards	4
CC6	V	Quarterly meetings without indicator-based monitoring	Mandate monthly audit-and-feedback cycles (hand hygiene, SSI rate, sterilisation quality, prophylaxis timing and device-associated infections)	5
CC5	S	Prevention measures regulated separately	Issue a single national SSI prevention bundle covering skin antisepsis, surgical hand preparation, prophylaxis timing, sterilisation assurance and operating-room discipline	6
CC3	V	Curriculum without competency assessment	Link course completion to a documented competency check	7
All	-	Alignment assessed from documents only	Conduct a national IPCAF-based facility assessment	8

CAUTI, catheter-associated urinary tract infection; CDC, Centers for Disease Control and Prevention; CLABSI, central-line-associated bloodstream infection; VAP, ventilator-associated pneumonia.

## Data Availability

The aggregated national HAI surveillance data analysed in this study are available from the corresponding author on request, owing to restrictions on redistribution by the State Sanitary-Epidemiological Surveillance Centres of the Republic of Uzbekistan. The regulatory texts reviewed (SanPiN No. 0342-17, SanPiN No. 0317-15, Order No. 92, SanQvaN No. 0020-22) are publicly available through the official channels of the Ministry of Health and the national legal information portal (lex.uz), which lists SanQvaN No. 0020-22 under its Ministry of Justice registration (No. 70, 4 March 2022). Methodical Guideline No. 06-11 is a technical guideline of the National Reference Laboratory rather than a registered normative act; it is available through the official channels of that Service (sanepid.uz) and from the corresponding author on request. Regional healthcare infrastructure indicators are publicly available from the Statistics Agency (stat.uz) [15]. The criteria and clause-level record underlying Table 4 are provided as Supplementary Tables S3 and S4.

## Supplementary Materials

The following supporting information can be downloaded at: https://www.mdpi.com/article/10.3390/healthcare14193161/s1, Table S1, Surveillance approaches for healthcare-associated infections: international definitions and current status in Uzbekistan; Table S2, Comparison of national HAI surveillance and IPC structures: Uzbekistan, Kazakhstan, and WHO recommendations; Table S3, Criteria and decision rule; Table S4, Clause-level record for all eight Core Components.

## Abbreviations

AAPC, average annual percentage change; AMR, antimicrobial resistance; CAUTI, catheter-associated urinary tract infection; CC, Core Component; CDC, Centers for Disease Control and Prevention (United States); CLABSI, central-line-associated bloodstream infection; DMED, national digital health information system of the Republic of Uzbekistan; ECDC, European Centre for Disease Prevention and Control; HAI, healthcare-associated infection; ICD-10, International Classification of Diseases, 10th revision; IPC, infection prevention and control; IPCAF, Infection Prevention and Control Assessment Framework; LMIC, low- and middle-income country; NHSN, National Healthcare Safety Network; SanPiN, Sanitary Rules and Norms; SSI, surgical site infection; VAP, ventilator-associated pneumonia; WHO, World Health Organization.

## References

[1] World Health Organization (2024). Health Care-Associated Infections Fact Sheet.

[2] World Health Organization (2020). Report on the Burden of Endemic Health Care-Associated Infection Worldwide.

[3] Allegranzi B., Bagheri Nejad S., Combescure C., Graafmans W., Attar H., Donaldson L., Pittet D. (2011). Burden of endemic health-care-associated infection in developing countries: Systematic review and meta-analysis. Lancet.

[4] Odoom A., Tetteh-Quarcoo P.B., Donkor E.S. (2025). Prevalence of hospital-acquired infections in low- and middle-income countries: Systematic review and meta-analysis. Asia Pac. J. Public Health.

[5] Klevens R.M., Edwards J.R., Richards C.L., Horan T.C., Gaynes R.P., Pollock D.A., Cardo D.M. (2007). Estimating health care-associated infections and deaths in U.S. hospitals, 2002. Public Health Rep..

[6] Murray C.J.L., Ikuta K.S., Sharara F., Swetschinski L., Aguilar G.R., Gray A., Han C., Bisignano C., Rao P., Wool E. (2022). Global burden of bacterial antimicrobial resistance in 2019: A systematic analysis. Lancet.

[7] World Health Organization (2024). Global Report on Infection Prevention and Control 2024.

[8] GBD 2021 Antimicrobial Resistance Collaborators (2024). Global burden of bacterial antimicrobial resistance 1990–2021: A systematic analysis with forecasts to 2050. Lancet.

[9] Viderman D., Khamzina Y., Kaligozhin Z., Khudaibergenova M., Zhumadilov A., Crape B., Azizan A. (2018). An observational case study of hospital associated infections in a critical care unit in Astana, Kazakhstan. Antimicrob. Resist. Infect. Control.

[10] Semenova Y., Yessmagambetova A., Akhmetova Z., Smagul M., Zharylkassynova A., Aubakirova B., Soiak K., Kosherova Z., Aimurziyeva A., Makalkina L. (2024). Point-prevalence survey of antimicrobial use and healthcare-associated infections in four acute care hospitals in Kazakhstan. Antibiotics.

[11] Smagul M., Yessmagambetova A., Semenova Y., Soiak K., Agazhaeva G., Zharylkassynova A., Yergaliyeva A., Aubakirova B. (2025). Prevalence of healthcare-associated infections and antimicrobial use in Kazakhstan: Results of the first nationwide survey in 2023. Antimicrob. Resist. Infect. Control.

[12] World Health Organization (2016). Guidelines on Core Components of Infection Prevention and Control Programmes at the National and Acute Health Care Facility Level.

[13] State Sanitary-Epidemiological Surveillance Centres of the Republic of Uzbekistan National Surveillance Reports on Healthcare-Associated Infections.

[14] National Statistics Committee of the Republic of Uzbekistan (2026). Permanent Population of the Republic of Uzbekistan, by Year (Official Annual Estimates).

[15] Statistics Agency under the President of the Republic of Uzbekistan (2024). Key Statistical Indicators of the Healthcare Sector of the Republic of Uzbekistan (as of 1 January 2024).

[16] Ministry of Health of the Republic of Uzbekistan (2017). Sanitary Rules and Norms No. 0342-17: Prevention of Healthcare-Associated Infections.

[17] Ministry of Health of the Republic of Uzbekistan (2015). Sanitary Rules and Norms No. 0317-15: Collection, Storage and Disposal of Waste from Healthcare Facilities.

[18] Ministry of Health of the Republic of Uzbekistan (2019). Order No. 92, 2 April 2019: On Approval of the Regulation on the Infection Control Committee in Treatment-and-Prophylactic Institutions and on Improving the Activity of the Committee (with Annexes 1-3).

[19] Sanitary-Epidemiological Welfare and Public Health Service of the Republic of Uzbekistan (2022). Resolution No. 0020-22, 21 January 2022: Sanitary Rules, Norms and Hygiene Standards for the Design, Construction and Operation of Treatment-and-Prophylactic Institutions (SanQvaN No. 0020-22).

[20] National Reference Laboratory, Sanitary-Epidemiological Welfare and Public Health Service of the Republic of Uzbekistan (2021). Methodical Guideline No. 06-11, 14 July 2021: Methods for Sanitary-Bacteriological Examination of Environmental Objects and Air, and Control of Sterility, in Medical Institutions.

[21] Saito H., Allegranzi B., Pittet D. (2018). 2018 WHO hand hygiene campaign: Preventing sepsis in health care and the path to universal health coverage. Lancet Infect. Dis..

[22] Jacobson J. (2018). Surgical site infection—The next frontier in global surgery. Lancet Infect. Dis..

[23] Mengistu D.A., Alemu A., Abdukadir A.A., Husen A.M., Ahmed F., Mohammed B., Musa I. (2023). Global incidence of surgical site infection among patients: Systematic review and meta-analysis. Inquiry.

[24] Allegranzi B., Aiken A.M., Kubilay N.Z., Nthumba P., Barasa J., Okumu G., Mugarura R., Elobu A., Jombwe J., Maimbo M. (2018). A multimodal infection control and patient safety intervention to reduce surgical site infections in Africa: A multicentre, before-after, cohort study. Lancet Infect. Dis..

[25] Peters S., Lim L.-L., Francis J.J., Bennett N., Fetherstonhaugh D., Buising K., McCahon J., Marshall C., Presseau J., Lim W.K. (2025). Analysis of infection prevention and control documentation in residential aged care based on a behaviour specification framework. Infect. Dis. Health.

[26] Tomczyk S., Storr J., Kilpatrick C., Allegranzi B. (2021). Infection prevention and control (IPC) implementation in low-resource settings: A qualitative analysis. Antimicrob. Resist. Infect. Control.

[27] Azak E., Sertcelik A., Ersoz G., Celebi G., Eser F., Batirel A., Cag Y., Ture Z., Engin D.O., Yetkin M.A. (2023). Evaluation of the implementation of WHO infection prevention and control core components in Turkish health care facilities: Results from a WHO IPCAF-based survey. Antimicrob. Resist. Infect. Control.

[28] Sreeramoju P., Song X., Bardossy A.C., Cadena J., A Forde C., Patel P., Salinas J., Tolliver C., Zayed B., Krein S.L. (2025). Infection prevention and healthcare epidemiology professionals in low- and middle-income countries: A needs assessment survey and call for action. BMJ Glob. Health.

[29] Ministry of Health of the Republic of Uzbekistan (2025). Order No. 270, 29 August 2025: On the Phased Introduction of the DMED (“Electronic Hospital”) Information System and Electronic Medical Record Forms in Treatment-and-Prophylactic Institutions.

[30] Murhekar M.V., Kumar C.P.G. (2022). Health-care-associated infection surveillance in India. Lancet Glob. Health.

[31] Tartari E., Tomczyk S., Pires D., Zayed B., Rehse A.C., Kariyo P., Stempliuk V., Zingg W., Pittet D., Allegranzi B. (2021). Implementation of the infection prevention and control core components at the national level: A global situational analysis. J. Hosp. Infect..

[32] Mwanja H., Waswa J.P., Kiggundu R., Mackline H., Bulwadda D., Byonanebye D.M., Kambugu A., Kakooza F. (2024). Utility of syndromic surveillance for the surveillance of healthcare-associated infections in resource-limited settings: A narrative review. Front. Microbiol..

[33] Coman A., Pop D., Muresan F., Oprescu F., Fjaagesund S. (2025). Surveillance and reporting of hospital-associated infections—A document analysis of Romanian healthcare legislation evolution over 20 years. Healthcare.

[34] Semenova Y., Akhmetova K., Rakhmatullaeva S., Muminova M., Fakhriddinova D., Dzhusupov K., Kanymetova A., Ashyralieva D., Saidova M., Yakubova S. (2026). Antibiotic resistance awareness and prescribing behavior among general practitioners in Kazakhstan, Kyrgyzstan, Uzbekistan, and Tajikistan. Antibiotics.

[35] Zingg W., Holmes A., Dettenkofer M., Goetting T., Secci F., Clack L., Allegranzi B., Magiorakos A.-P., Pittet D. (2015). Hospital organisation, management, and structure for prevention of health-care-associated infection: A systematic review and expert consensus. Lancet Infect. Dis..

[36] Bunduki G.K., Masoamphambe E., Fox T., Musaya J., Musicha P., Feasey N. (2024). Prevalence, risk factors, and antimicrobial resistance of endemic healthcare-associated infections in Africa: A systematic review and meta-analysis. BMC Infect. Dis..

[37] World Health Organization (2018). Infection Prevention and Control Assessment Framework (IPCAF) at the Facility Level.

[38] Asgedom A.A. (2024). Status of infection prevention and control (IPC) as per the WHO standardised Infection Prevention and Control Assessment Framework (IPCAF) tool: Existing evidence and its implication. Infect. Prev. Pract..

[39] Li L., Leng K., Du X., Wang G., Liu M., Meng Q. (2026). Evaluating infection prevention and control implementation in hospitals of underdeveloped region of China using the standardized WHO-IPCAF tool. Front. Public Health.

