# Supplementary material for: National Trends in Healthcare-Associated Infections and Evaluation of Infection Prevention and Control Policies in Uzbekistan (2015–2024)
Source: Healthcare (Basel). 2026 Sep 24;14(19):3161. doi: 10.3390/healthcare14193161 (PMC13665068; doi:10.3390/healthcare14193161)
Supplement: Supplementary file 1 [file healthcare-14-03161-s001.zip › healthcare-4527453-supplementary.pdf]

MDPI

healthcare

Supplementary Material

National Trends in Healthcare-Associated Infections and Evaluation of Infection Prevention and Control Policies in Uzbekistan (2015–2024)

This supplementary material accompanies the main manuscript. Tables S1 and S2 provide international context for the surveillance and monitoring/audit comparison discussed in the Discussion (CC4, CC6). Tables S3 and S4 document the coding criteria and the complete clause-level record underlying Table 4 of the main text, so that every classification can be independently reproduced or contested.

Table S1. Surveillance approaches for healthcare-associated infections: international definitions and current status in Uzbekistan

Surveillance approach	Core definition	Typical case-finding method	International reference example	Current status in Uzbekistan (per reviewed regulatory texts)
Passive surveillance	Reliance on routine clinical reporting by treating staff, without dedicated case-finding personnel	Spontaneous notification by ward staff using standard forms	Traditional hospital infection-control reporting in many LMIC settings	Predominant model; based on mandatory notification form F.085/U and ICD-10 coded logbooks
Active surveillance	Systematic, planned case-finding by dedicated infection-control personnel using standardised criteria	Scheduled chart review, laboratory-result review, or ward rounds by IPC staff	CDC/NHSN-style active surveillance in acute-care hospitals	Not described as a distinct, codified requirement in the regulatory texts reviewed
Electronic surveillance	Automated or semi-automated case detection using electronic health record, laboratory, or pharmacy data	Algorithm-assisted flagging of likely HAI cases from digital data sources	Electronic algorithms integrated into hospital information systems in high-income settings	No dedicated electronic HAI surveillance module identified; the national digital health platform (DMED) is being rolled out for broader clinical records but, per Order No. 270 (2025), has not been extended to HAI-specific case detection,

 https://doi.org/10.3390/healthcare14193161

				confirmation, or registry functions
AI-assisted surveillance	Machine-learning models applied to structured/unstructured clinical data to predict or flag probable HAI cases	Predictive algorithms trained on electronic health record or laboratory data streams	Emerging pilot applications in some high-income health systems; not yet standard practice internationally	Not implemented or referenced in the regulatory texts reviewed; identified here as a longer-term direction rather than a current gap
ECDC point-prevalence survey (PPS) methodology	Standardised, periodic cross-sectional survey of all inpatients on a single day using harmonised case definitions	Trained surveyors examine patients and records against ECDC HAI case definitions	ECDC PPS, applied regionally in the Kazakhstan nationwide survey (2023)	Not yet applied in Uzbekistan; no comparable point-prevalence survey identified in the literature reviewed
US CDC/NHSN surveillance definitions	Standardised, continuous, facility-level surveillance using NHSN case definitions and risk-adjusted reporting	Continuous, protocol-driven case ascertainment against NHSN definitions, often electronically supported	US National Healthcare Safety Network (NHSN)	Not adopted as the basis for national case definitions; current definitions follow ICD-10 coding rather than NHSN-equivalent criteria

Table S2. Comparison of national HAI surveillance and IPC structures: Uzbekistan, Kazakhstan, and WHO recommendations

Domain	Uzbekistan (current national framework)	Kazakhstan (per published surveys)	WHO recommendation / Core Component
HAI surveillance model	Passive, paper-based notification (Form F.085/U) with retrospective ICD-10 coding; no dedicated electronic HAI module	ECDC point-prevalence survey methodology applied nationally in 2023 (26 hospitals, 8,076 patients)	CC4: standardised, ideally active and/or electronic, continuous surveillance using internationally harmonised case definitions
Staffing (dedicated epidemiologist/IPC professional)	Mandatory only in facilities with >200 beds; discretionary below this threshold. This provision is classified under CC1 (programme staffing, universality criterion not met) and is not counted towards CC7, whose subject matter — nurse staffing, workload, and bed occupancy — has no corresponding provision	Not systematically reported in the published surveys reviewed; IPC staffing assessed qualitatively in pilot studies	CC1/CC7: a trained IPC professional or team present at every acute-care facility regardless of size, with adequate nurse staffing and bed-occupancy standards

Electronic surveillance / digital health integration	National digital health platform (DMED) in progressive rollout; per Order No. 270 (2025), not yet extended to HAI-specific case detection, confirmation, or registry functions	Not the primary methodology in the surveys reviewed (survey-based rather than continuous electronic surveillance)	Encouraged as a long-term direction to support real-time, continuous CC4 implementation
Monitoring, audit and feedback	Infection Control Committee formally mandated at every facility regardless of size, meeting at least quarterly, with Methodical Guideline No. 06-11 specifying sanitary-bacteriological monitoring and sterility-control methods; no indicator-based feedback cycle or mechanism verifying that committees function as intended	Not directly comparable; point-prevalence surveys provide periodic, not continuous, monitoring	CC6: continuous monitoring and regular, structured feedback to facilities and staff
Built environment, materials, waste management	Detailed five-class medical waste classification and colour-coding (SanPiN No. 0317-15), with quantitative room air-cleanliness classes (CFU/m³), air-exchange rates, wet-cleaning frequency, and sterilisation-department zoning (SanQvaN No. 0020-22), verified through Methodical Guideline No. 06-11	Not the focus of the surveys reviewed	CC8: adequate built environment, materials, and equipment to support IPC at the point of care
Most recent national-level HAI data source	National annual surveillance statistics, 2015–2024 (population-based incidence and, for 2024, region-specific incidence)	Nationwide ECDC-methodology point-prevalence survey, 2023 (facility-based prevalence)	WHO recommends both routine surveillance and periodic point-prevalence surveys as complementary tools

Table S3. Criteria and decision rule

This table sets out in full the criteria summarised in the Materials and Methods. For each WHO Core Component, all corresponding provisions across the five national instruments were identified and evaluated against the five binary criteria below. The decision rule then maps the criterion profile onto a category directly; no global or impressionistic judgement is involved at the classification step.

Criteria

https://doi.org/10.3390/healthcare14193161

Code	Criterion	Question asked of the identified provision(s)
S	Corresponding scope	Does a provision address the Core Component as WHO defines it, including any integrative requirement (e.g., CC5 requires a coordinated multimodal strategy, not merely separate regulation of its elements)?
M	Mandatory	Is the provision legally binding rather than discretionary, advisory, or recommendatory?
Q	Quantified	Does the provision state a measurable requirement — a minimum frequency, threshold, duration, ratio, or numerical standard — rather than a general aspiration?
U	Universal	Does the provision apply to all relevant facilities, rather than only to those above a size, tier, or bed threshold?
V	Verification	Does a monitoring, audit, or reporting mechanism exist by which compliance with the provision can be checked?

Decision rule

Category	Rule
Full	All five criteria (S, M, Q, U, V) met.
Partial	M met, together with at least one but not all of S, Q, U, V.
Weak	No corresponding provision exists; or the only identified provisions are discretionary (M not met); or the substantive content of the Core Component is left unaddressed.

Application notes

1. The unit of analysis is the individual regulatory provision (article, clause, or annex item), not the instrument as a whole.
2. A provision is counted under one Core Component only, according to its subject matter. Where a provision could arguably serve two Components, it is assigned to the Component whose definition it matches most directly; the reason is recorded in Table S4. This rule was introduced during joint re-adjudication and is the reason the 200-bed epidemiologist threshold is counted under CC1 (programme staffing) and not under CC7 (nurse staffing, workload, bed occupancy).
3. Criterion V is assessed against an explicit mechanism in the regulatory text. The existence of general sanitary-epidemiological inspection powers was not treated as satisfying V, because those powers are not tied to the specific provision being assessed.

Coding process. Coding was performed independently and blinded by two authors (N.T.K., B.B.R.). In the independent round the coders agreed on 6 of 8 components (75% observed agreement; Cohen's $\kappa = 0.53$, 95% CI −0.04 to 1.00, truncated at the upper bound), with both disagreements (CC1, CC3) between adjacent categories. All eight components were then re-adjudicated jointly against the criteria, recording the clause relied upon and the outcome on each criterion (Table S4). This round resolved both disagreements and revised two previously agreed components (CC5 from Full to Partial; CC7 from Partial to Weak), so final classifications differ from the initial independent scores for four of eight components (CC1, CC3, CC5, CC7). The κ statistic therefore describes the initial coding round and the adequacy of the criteria wording at that stage; it is not a precision estimate for the final classifications, which should instead be assessed against the record in Table S4.

https://doi.org/10.3390/healthcare14193161

Table S4. Clause-level record for all eight Core Components

Each row records the WHO requirement, the corresponding national provision(s) identified, the outcome on each criterion with the reason where the criterion is not met, and the resulting category under the decision rule in Table S3. Readers who dispute a classification can identify the specific criterion and clause in question.

CC	WHO requirement (summary)	Provision(s) identified	S	M	Q	U	V	Reason for any criterion not met	Category
CC1	IPC programme with trained IPC team at national and facility level	Order No. 92 (2019), Regulation on the Infection Control Committee and Annexes 1–3: committee mandatory in every facility; dedicated hospital epidemiologist mandatory only above 200 beds	✓	✓	✓	✗	✗	U: dedicated IPC professional not required below 200 beds. V: no mechanism verifying that the programme functions as specified	Partial
CC2	Evidence-based IPC guidelines, implemented and periodically updated	SanPiN No. 0342-17: comprehensive national IPC guideline in force since 2017	✓	✓	✓	✓	✗	V: no scheduled revision cycle and no audit of adherence specified within the instrument	Partial
CC3	IPC education and training with evaluation of effectiveness	Order No. 92 (2019), Annex on staff training: mandatory induction and periodic training; 12-topic, 36-hour curriculum (3 h per topic) for physicians and mid-level staff	✓	✓	✓	✓	✗	V: no post-training competency assessment or evaluation of training effectiveness specified	Partial
CC4	Facility-based surveillance using standardised case definitions, guiding IPC action	SanPiN No. 0342-17: mandatory emergency notification (Form F.085/U) for each suspected case, ICD-10 coding by the Surveillance Centres	✗	✓	✗	✓	✗	S: passive notification only; no standardised case definitions, active case-finding, or denominators. Q: no stated case definition, frequency, or coverage standard. V: no data-quality audit or completeness check	Partial
CC5	Multimodal strategies implementing IPC activities as one coordinated approach	SanPiN No. 0342-17: hand hygiene, disinfection, sterilisation, and isolation regulated in separate chapters	✗	✓	✓	✓	✗	S: elements regulated individually; no coordinated multimodal strategy required. V: no compliance monitoring (e.g., hand hygiene observation) specified	Partial
CC6	Regular monitoring and	Order No. 92 (2019): Infection Control	✓	✓	✓	✓	✗	V: no indicator-based feedback cycle and no	Partial

https://doi.org/10.3390/healthcare14193161

	audit with timely feedback to staff	Committee mandatory in every facility, meeting at least quarterly, extraordinary meetings permitted; Methodical Guideline No. 06-11 (2021): sanitary-bacteriological monitoring and sterility-control methods						mechanism verifying that committees perform the monitoring function	
CC7	Adequate nurse staffing, bed occupancy of one patient per bed with adequate spacing, workload standards	None identified. The 200-bed epidemiologist threshold is counted under CC1 (see Table S3, application note 2)	✗	✗	✗	✗	✗	No corresponding provision for nurse staffing, workload, or bed occupancy in any of the five instruments	Weak
CC8	Built environment, materials and equipment adequate for IPC at the point of care	SanPiN No. 0317-15: five hazard classes (A–D) with colour-coding, packaging, decontamination; SanQvaN No. 0020-22: room air-cleanliness classes (CFU/m^3), air-exchange rates, wet-cleaning frequency, sterilisation-department zoning; Methodical Guideline No. 06-11: sanitary-bacteriological and sterility monitoring methods	✓	✓	✓	✓	✓	–	Full

Summary of criterion outcomes. Verification (V) was the criterion most frequently unmet (7 of 8 components), followed by corresponding scope (S; 3 of 8), universality (U; 2 of 8), and quantification (Q; 2 of 8). Mandatory status (M) was met for every component with an identified provision.

https://doi.org/10.3390/healthcare14193161
